# Development of a core outcome set for IBD patients with stomas

**DOI:** 10.1093/ecco-jcc/jjag157

**Published:** 2026-10-28

**Authors:** Lakshman Kumar, Micheal Hanly, Anne Fennessy, Neil O’Morain, Sean Martin, Aine Keogh, Richéal Burns, Amber Kehoe, Nicola Dames, Merel Kimman, Kapil Sahnan, Phil Tozer, Glen Doherty, TOAST-IBD Collaborators, Colm O’Morain, Aoibhlinn O’Toole, Eoin Slattery, Orlaith Kelly, Cara Dunne, Catherine Rowan, Jayne Doherty, Roisin Stack, Karl Hazel, Piotr Eder, Mark Samaan, Christian Sellinger, Michael Kelly, John Burke, Audrius Dulskas, Jasper Stijns, Eleni Andriopoulou, Mary Hamzawi, Carolann Coe, Joanna Rea, Catherine Walsh, Marie Grimes, Susan Moore, Wladzia Czuber-Dochan, Kathleen Cafferky, Caron Allan, David MacDougald, Dag Stockstad, Martina O’Grady

**Affiliations:** Department of Gastroenterology, St. Vincent’s University Hospital, Dublin, D04 T6F4, Ireland; School of Medicine, University College Dublin, Dublin, Ireland; Department of Gastroenterology, St. Vincent’s University Hospital, Dublin, D04 T6F4, Ireland; School of Medicine, University College Dublin, Dublin, Ireland; Department of Gastroenterology, St. Vincent’s University Hospital, Dublin, D04 T6F4, Ireland; School of Medicine, University College Dublin, Dublin, Ireland; Department of Gastroenterology, St. Vincent’s University Hospital, Dublin, D04 T6F4, Ireland; School of Medicine, University College Dublin, Dublin, Ireland; Department of Colorectal Surgery, St. Vincent’s University Hospital, Dublin, Ireland; Department of Gastroenterology, University Hospital Galway, Galway, Ireland; Department of Health and Nutritional Science, Atlantic Technological University, Sligo, Ireland; Patient Representative Group, Investigator Network for Inflammatory Bowel Disease Therapy in Ireland (INITIative), Dublin, Ireland; Patient Liaison Group (PLG), Association of Coloproctology Great Britain & Ireland (ACPGBI), Glasgow/Oxford, United Kingdom; Department of Clinical Epidemiology and Medical Technology Assessment (KEMTA), Maastricht University Medical Centre, Maastricht, The Netherlands; Department of Colorectal Surgery, St Mark’s the National Bowel Hospital and Academic Institute, London, United Kingdom; Department of Colorectal Surgery, St Mark’s the National Bowel Hospital and Academic Institute, London, United Kingdom; Department of Gastroenterology, St. Vincent’s University Hospital, Dublin, D04 T6F4, Ireland; School of Medicine, University College Dublin, Dublin, Ireland; Investigator Network for Inflammatory Bowel Disease Therapy in Ireland (INITIative), Dublin, Ireland

**Keywords:** inflammatory bowel disease, ostomy, core outcome set

## Abstract

**Background and Aims:**

A significant number of patients with inflammatory bowel disease (IBD) undergo stoma-forming surgery. For many, an ostomy is not curative, and disease exacerbations requiring ongoing therapy may be required. Despite this, IBD patients with stomas are frequently excluded from clinical trials due to the heterogeneity in outcomes and the absence of validated measurement tools. The TOAST-IBD project aimed to develop a Core Outcome Set (COS) for IBD patients with stomas undergoing interventional trials to address this issue.

**Methods:**

Candidate outcomes were identified through a systematic literature review and patient focus groups. These were refined using a two-round online Delphi process, with scoring based on the importance of each outcome in determining treatment success. Participants were recruited internationally and grouped into four stakeholder panels: patients, specialist and research nurses, colorectal surgeons, and gastroenterologists. Feedback was provided after each round to allow score refinement. A final consensus meeting determined the COS.

**Results:**

A total of 81 outcomes were identified and categorized into five themes. In total, 76 stakeholders (73.1% response rate) from 17 countries participated in the Delphi process, carrying 37 outcomes forward. At the final consensus meeting, 37 experts and patients agreed on a six-domain COS: maintenance of hydration and volume status, maintenance of nutritional status, symptom-based clinical activity, objective marker-based disease activity, and two quality-of-life domains (IBD-related and stoma-specific).

**Conclusion:**

An IBD Stoma COS has been produced by all key stakeholders. This COS will standardize outcome reporting and support creation of validated measurement instruments, facilitating the inclusion of this underserved group in future clinical trials.

**Registration:**

This study has been registered with the COMET Initiative as study 2855 (https://www.comet-initiative.org/Studies/Details/2855) and followed its recommended methodological procedures. The systematic review has been registered with the PROSPERO database (PROSPERO-ID: CRD42023442388).

## Graphical abstract

**Graphical Abstract:**
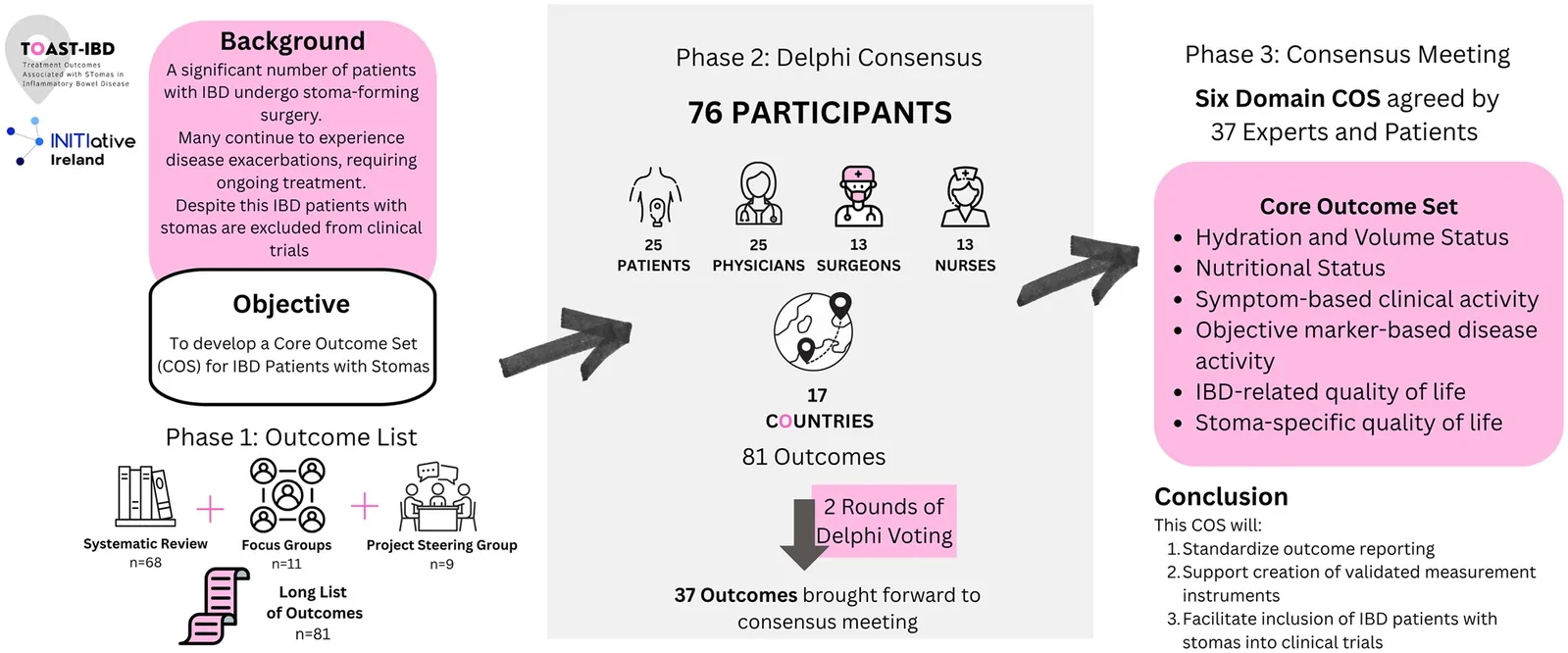

## 1. Introduction

Crohn’s disease (CD) and ulcerative colitis (UC) are collectively known as inflammatory bowel disease (IBD) and a small number of patients are diagnosed with IBD-unspecified, where a distinction between the two conditions is not possible. They are chronic relapsing–remitting diseases of the gastrointestinal tract, with peak age of onset in children and young adults.^1^ There is currently no cure and the diseases are associated with lifelong morbidity. The majority of patients with CD and many patients with UC require surgical resection as part of their treatment.^2^^,^^3^ Of these patients, many will need either a temporary or permanent stoma following surgery.^4^^,^^5^ Although many patients with an ostomy report overall improved quality of life following surgery, studies have shown that those who had undergone surgery (either stoma-forming or non-stoma-forming) experience more body image dissatisfaction and higher rates of sexual dysfunction.^6^^,^^7^ For a considerable number of patients with CD, surgery requiring an ostomy is not curative and patients may continue to experience disease exacerbations and require continued medical therapies.^8^

The Core Outcome Measures in Effectiveness Trials (COMET) initiative aims to improve understanding of outcome reporting and standardized outcome reporting through the development of guidelines for Core Outcome Set (COS) development.^9^ A COS is a set of the minimum outcomes that should be reported in every study of a given condition. They have usually been informed by a systematic review and developed through a Delphi consensus process with key stakeholders.^9^ COSs are not restrictive; triallists may choose to investigate other outcomes but should always include the COS as a minimum within their study design. Patients with stomas are excluded from clinical trials of investigational medicinal products,^10^ primarily because of the difficulty in calculating disease activity scores with measures such as the Crohn’s Disease Activity Index (CDAI) and the Mayo Score for UC. Improvement in these disease activity indices (DAIs) are typically used as the primary endpoint for clinical trials to determine response to drug therapies and other interventions for IBD.^11^ Dedicated COSs have recently been developed for IBD^12^ as well as for fistulizing peri-anal CD.^13^ A similar approach is needed in IBD patients with stomas, especially for patients with CD, who often have treatment refractory disease, have failed medical therapies, and need access to new therapies. TOAST-IBD is a collaborative project established by INITIative (www.initiativeibd.ie), the IBD research network for Ireland, to determine the most relevant outcomes that can be used to assess the clinical effectiveness of medical, surgical, nutritional, physical, and psychological treatment inventions for patients with IBD who have stomas.

## 2. Aim

To develop a COS following COMET guidelines for use in intervention trials for adults with IBD who have a stoma using the Delphi consensus methodology and an international consensus meeting.

## 3. Scope

This COS is being developed for:

- Adults (≥18 years) diagnosed with IBD who have a stoma of their bowel. This includes all subtypes of stomas permanent or temporary, including ileostomies and colostomies.
- All interventions. This will include, but not be limited to, pharmacological, surgical, psychological, and dietary interventions.
- For use in clinical effectiveness research, investigating treatment for IBD patients with stomas.

## 4. Methods

### 4.1. Phase 1—developing a long list of candidate outcomes

#### 4.1.1. Systematic literature review

A systematic literature review was performed to identify a comprehensive list of candidate outcomes that have been measured in trials of interventions for patients with IBD. These candidate outcomes were then categorized into distinct groups of patient-reported and clinician-reported outcomes. Support for the literature review was provided by the University College Dublin (UCD) Clinical Research Centre with access to dedicated research librarians.

A systematic review of studies assessing medical, surgical and combined (medical and surgical) treatment of patients with CD and UC who have stomas was performed in accordance with a prospectively registered protocol. The OVID SP version of MEDLINE (1950-2023), Embase (1980-2023), CENTRAL (1996-2023), and PsycINFO (2013-2023) was searched using validated terms for relevant concepts: “Inflammatory bowel disease,” “stoma/ostomy” and “randomized controlled trials or prospective studies.” Within each concept, terms were separated using the Boolean operator “OR”—for example “Crohn’s disease” OR “Ulcerative colitis” OR “Inflammatory bowel disease” and these concept groups were combined by the Boolean operator “AND.” This was supplemented with a free-text search of these databases, using relevant keywords/terms (including synonyms and variants), also combined by the Boolean operator “AND.” The search was limited to studies conducted in human adults aged ≥18 years old and to papers published between January 1, 2001 and December1, 2023, in order to ensure that identified outcomes were contemporary and currently applicable. Prospective studies (including cohort comparisons, case controls, and case series) that reported on outcomes on an intervention (medical, surgical, nutritional, or any combination) for patients with IBD who have stomas and recruited ≥10 patients were included. Systematic reviews of clinical trial outcomes in IBD were included, and the individual studies reviewed were searched to ensure complete capture. Studies were excluded where stoma surgery was performed in patients who did not have IBD or were not published in the English language.

Output from the search was imported into Covidence, a web-based software platform, which was used to screen the generated studies. Two independent reviewers (LK, MH) used predefined selection criteria to screen the studies, and each study was reviewed by at least two independent reviewers. Studies were initially screened in abstract format before full-text review. Conflicts were resolved by discussion with recourse to a third reviewer (GD) where necessary. Extracted data included study publication year, study design, type of intervention, patient information (ie, number of patients, disease subtype, type of stoma studied), outcomes (primary and secondary), outcome definitions, and measurement instruments used. In keeping with COMET recommendations, all data were extracted verbatim.^14^

Three members of the study team, one physician (LK), one nurse researcher (AK), and a patient researcher (ND), categorized outcomes identified from the systematic review into domains by consensus according to the taxonomy for outcomes defined by COMET.^15^ All the included outcomes were categorized into themes and presented as such throughout the rest of the process. Discrepancies were resolved through discussion with the other reviewers.

#### 4.1.2. Patient focus groups

There are little published data on the patient’s perspective of living with IBD and having a stoma, especially if this represents non-curative/non-definitive treatment. We aimed to understand their experience of living with a stoma and its impact on daily life and routine functioning. An exploratory qualitative study was conducted using purposive sampling to recruit participants with an IBD diagnosis living with a stoma. The “standards for reporting qualitative research” (SRQR) recommendations^16^ were followed. Semi-structured focus group interviews were conducted via an online platform, during which the audio was recorded, transcribed verbatim, and translated (if necessary) to be analyzed thematically. A member of the steering group (RB) who has extensive experience in qualitative research directed the series of semi-structured interviews with patients. Early themes were reviewed by the Project Steering Group (PSG) and the outcomes identified from these focus groups that were not captured in the systematic review were added to the “long list” of outcomes. The full results of this qualitative study have been submitted for publication.

#### 4.1.3. Finalizing “long list” of outcomes

The project steering group, consisting of all stakeholder groups and methodologists, reviewed and finalized the domains and list of outcomes. We created plain English language definitions for all outcomes under consideration. The final “long list” of all possible outcomes were presented to the stakeholders through a web-based system designed to conduct a three-phase Delphi process.

### 4.2. Phase 2—Delphi consensus

Candidate outcomes were presented via a web-based Delphi system. Methodological support for this process was obtained from the national TMRN (Trials Methodology Research Network, Ireland).

#### 4.2.1. Stakeholder panels

Four stakeholder panels (medical, surgical, nursing, and patient panels) were formed recognizing that patients with IBD who have stomas are managed by multidisciplinary teams, including primary care practitioners, surgeons, gastroenterologists, IBD specialist nurses, and stoma care nurse specialists.

#### 4.2.2. Recruitment

All stakeholders were recruited through collaborating organizations with the project steering group working to facilitate the process of ensuring equal and broad representation. Patients were recruited through the Irish Society of Crohn’s and Colitis (IBD patient representative association) and the Ostomy Association of Ireland (representative association for patients living with a stoma/ostomy). Nurses were recruited through the established collaboration with the IBD Nurse Association of Ireland (IBD specialist nurses representative association) and the Irish Stoma Care and Colorectal Nurses Association. Physicians were recruited through INITIative (IBD collaborative research network for Ireland) and engagement with the Irish Society for Gastroenterology (ISG) and Irish Association of Coloproctology (IACP). In order to improve the social, geographic, economic, and cultural applicability of the process, health professionals and patients were recruited in Europe, Asia, and North America by directly contacting the respective regional representative associations for each discipline (eg, the European Crohn’s and Colitis Organisation and the European Society of Coloproctology). Recruitment for the study was also conducted via social media, specifically X (formerly Twitter), to increase overall reach to international counterparts.

A participant information sheet was made available on the webpage of the each of the collaborating organizations explaining the project and seeking expression of interest. On registration, interested participants were asked to provide information through an online survey and invited to complete an initial registration survey to capture core demographic data and ensure gender, age, and geographic balance ensuring that each stakeholder panel was representative. The patient panel featured patients with both ileostomy and colostomy (both temporary/loop and permanent ostomies). To maximize ongoing commitment to the process, we offered acknowledgement of all (consenting) participants completing all three rounds on publication of the study and sent newsletters after each of the Delphi rounds. Stakeholders were given the option to withdraw from the project at any time either by contacting the PSG or also by simply not responding to emails/questionnaires.

Consent from participants in the online survey was sought online prior to accessing the survey, along with screening questions to confirm eligibility. Participants were reminded that they were free to withdraw from the survey at any time without giving a reason, and could request that their survey responses are withdrawn from analysis, if not already presented. There was no restriction on the number of eligible participants and we aimed to recruit equal numbers from each relevant discipline.

#### 4.2.3. Online Delphi consensus

A two-round online Delphi process was used to prioritize outcomes. In each round, participants scored outcomes using the numerical scale suggested by the Grading of Recommendations Assessment, Development and Evaluation (GRADE) group (http://www.gradeworkinggroup.org).^17^ Scale 1-3 signified an outcome of limited importance (categorized as “not important”), 4-6 signified important but not critical (categorized as “fairly important”), and 7-9 signified an outcome of critical importance (categorized as “really important”). In addition, a free-text space was provided for stakeholders to comment on the outcome definitions and qualify their responses.

Outcomes were classified according to predefined consensus criteria (Table 1). Outcomes were carried forward between rounds if >70% of all participants scored them as “really important.” Outcomes where <70% of clinicians and clinical researchers rate as “7-9” but average >7 in the patient panel were considered to be “patient-important” and were classified as “consensus in.” Outcomes where <70% of clinicians and clinical researchers rate as “7-9” and average <7 in the patient panel were classified as “consensus out.” All remaining outcomes were classified as having achieved “no consensus.” Only outcomes classified as “consensus in” were carried forward to the next round.^18^ These criteria distinguished outcomes with insufficient support across both clinical and patient stakeholder groups (“consensus out”) from outcomes with discordant or borderline ratings (“no consensus”), providing transparency for voters participating in the next round and consensus meeting.

**Table 1.** Classification and definition of outcomes.

Consensus classification	Definition	Description
**Consensus in**	>70% of participants in each panel rate the outcome “7-9” OR <70% of clinicians and (clinical) researchers rate the outcome “7-9” AND an average patient rating of >7	Important outcome
**Consensus out**	<70% of the clinicians and (clinical) researchers rate the outcome “7-9” AND an average patient rating of <7	Not important outcome
**No consensus**	All other outcomes that do not meet the above criteria	Somewhat important outcome

In the first round, participants were asked to score each outcome based on how important they rated it in evaluating the effect of an intervention (eg, pharmacological, dietary, surgical, psychological) and to suggest additional outcomes they felt were important but which were not represented in the first “long list.” Newly suggested outcomes were reviewed by the project steering group and taken forward for evaluation in the next round if it was judged they fall within the scope of the COS. Participants who completed the first round were eligible to take part in the second round.

In the second round, participants were asked to score the “consensus in” outcomes again having been shown numerical and graphical representations demonstrating how others in their own panel scored each outcome in the initial round as well as how others in each of the other panels scored each outcome (compared to their own response from the first round if applicable). Participants who completed the second round were eligible to take part in the third round (if required).

In this study, following a discussion with the project steering group, a third round was not conducted as there was sufficient consensus achieved in the first two rounds. Similar to the second round, participants would rescore the outcomes having been shown numerical and graphical representations of how all panels scored outcomes in the previous round. They were also shown their response from the prior rounds. Only participants who completed all rounds of voting were invited to take part in the final consensus meeting.

Each round was open for 4 weeks. The PSG assessed outcomes for heterogeneity between the stakeholder panels (using a histogram depicting median scores), at each stage of the process. Comments on the clarity and appropriateness of each outcome from each round were also reviewed by the study management group and outcomes modified to improve clarity if necessary.

#### 4.2.4. Reducing attrition

To reduce the number of participants not completing the surveys, a reminder email was sent to participants who had not completed the round after 2 weeks and then again at 3 weeks. Video newsletters were sent to all participants between each round to update them on progress and modifications, in addition to social media promotion to encourage their continued participation in the process.

#### 4.2.5. Final consensus

A consensus meeting with representatives of all the stakeholders was conducted to finalize the COS. The outcomes that were prioritized during the Delphi process were discussed and voted on at an online consensus meeting. Electronic voting was used to maintain anonymity. An initial vote, “Agree,” “Do not agree,” or “Unsure,” was followed by debate among participants, refinement of the wording of the consensus statement and then a second vote. Outcomes where ≥70% of participants who voted for their inclusion was retained in the final COS.

The overall study design and methodological workflow are summarized in Figure 1.

**Figure 1.**
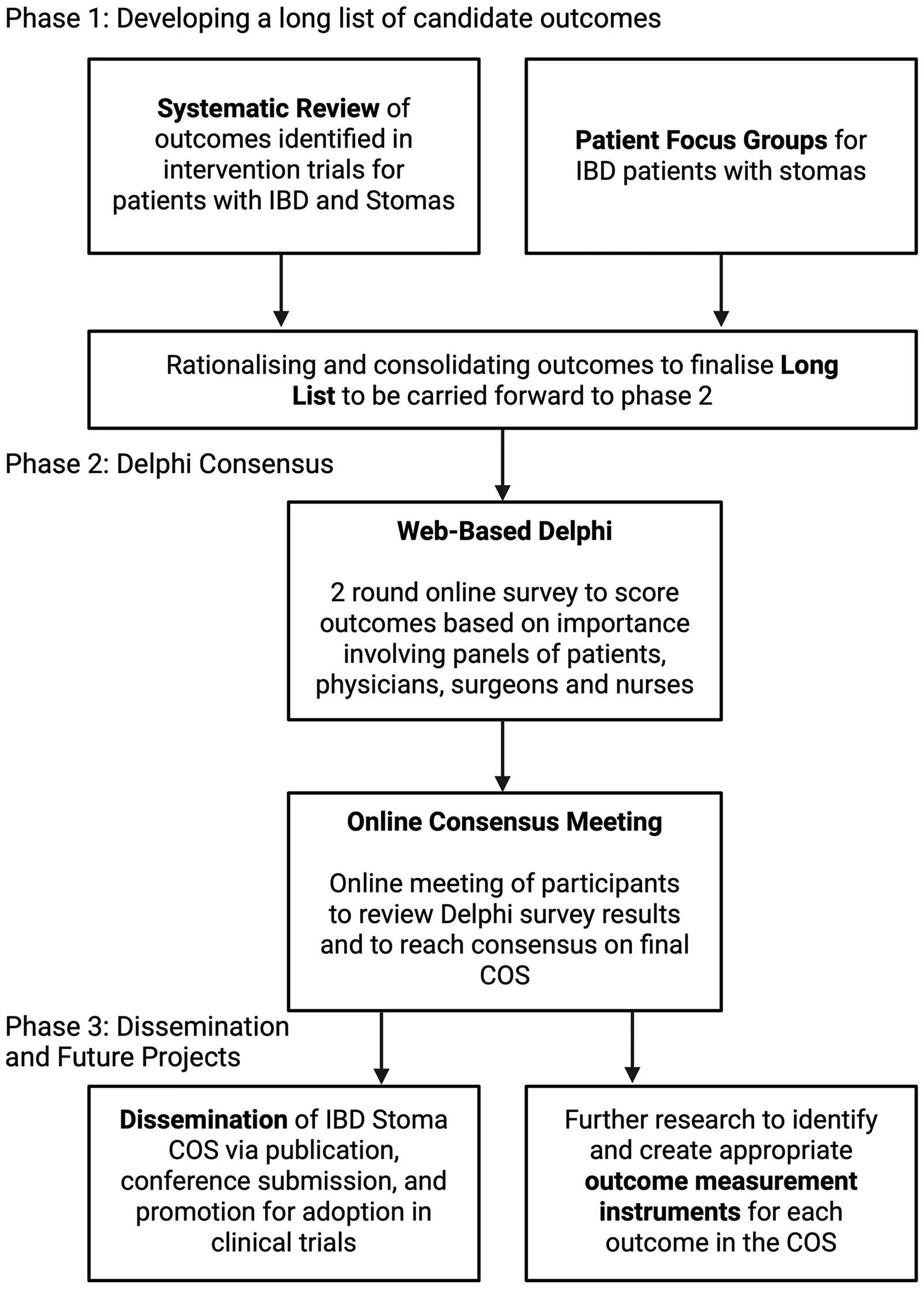
TOAST-IBD flowchart.

### 4.3. Phase 3—dissemination of the COS and future research

Results of this COS were disseminated to all participants involved in its creation. Furthermore, these results will be passed on to regulatory bodies, clinical trial registries, as well as pharmaceutical companies involved in clinical research to encourage adoption of the COS in future studies. Leading on from this, the focus of research stemming from this will aim to explore and validate the outcome measurement instruments needed for each of the agreed upon outcomes (ie, which tools or scoring systems do we use to measure the constituents of the COS?)

### 4.4. Patient and public involvement

Patients were involved in the design and conduct of this project from an early stage. Two patient representatives were members of the PSG and contributed to the oversight, protocol refinement, and methodological decisions throughout the project. The priority of the research question was informed by discussions with patients through structured online focus groups and engagement with national IBD and ostomy advocacy organizations. Patients also contributed to the choice of outcomes by identifying additional outcomes not captured in the systematic review, which were incorporated into the Delphi process. They participated as a dedicated stakeholder panel in the Delphi consensus and were represented at the final consensus meeting, contributing equally through structured discussion and anonymous voting.

### 4.5. Ethical considerations

Ethical approval was obtained prior to commencement from the St. Vincent’s Hospital Group Research Ethics Committee (Ref. No.: RS22-037). All participants provided informed consent prior to participation. For the online Delphi process, consent was obtained electronically before access to the survey platform. Participants in the patient focus groups provided written informed consent before interviews were conducted. Participants were informed that their involvement was voluntary, that they could withdraw at any time without providing a reason, and that their data would be anonymized prior to analysis. Data were stored securely in password-protected institutional systems and handled in accordance with applicable data protection regulations. Only authorized members of the PSG had access to identifiable data during the conduct of the study.

All authors had access to the study data and reviewed and approved the final manuscript.

## 5. Results

### 5.1. Participants

A total of 141 participants registered initial interest. Of these, 104/141 registered their demographics and completed round 1 of the Delphi Survey. In total, 76 participants (73.1%) from 17 countries (including participants from Europe, Asi,a and North America) then went on to complete round 2. In total, 37 participants attended an online consensus meeting across the panels (25 patients, 25 gastroenterologists, 13 colorectal surgeons, and 13 academic and specialist nurses [eg, IBD specialist and stoma therapy nurses]). Participant details are summarized in Supplementary File 1.

### 5.2. Information sources and domain categorization

A total of 1063 titles were identified from the systematic review, of which 325 were duplicates and the remaining 738 were screened. Of these, a further 665 studies were excluded based on title and abstract review. Following full text review, a further 58 studies were excluded for the following reason: only the abstract was available, non-prospective study design, insufficient patient recruitment, no intervention included. This resulted in 15 included studies in the final analysis.^19–33^ No additional papers were identified from systematic reviews or other trials. In total, eight were prospective observational studies, four were randomized control trials, and three were systematic reviews and meta-analyses. Four studies assessed patient quality of life, three studies were exclusive to IBD patients with stomas, and no studies included the use of small molecules or biologics. The study selection process is summarized in the PRISMA flow diagram (Figure 2).

**Figure 2.**
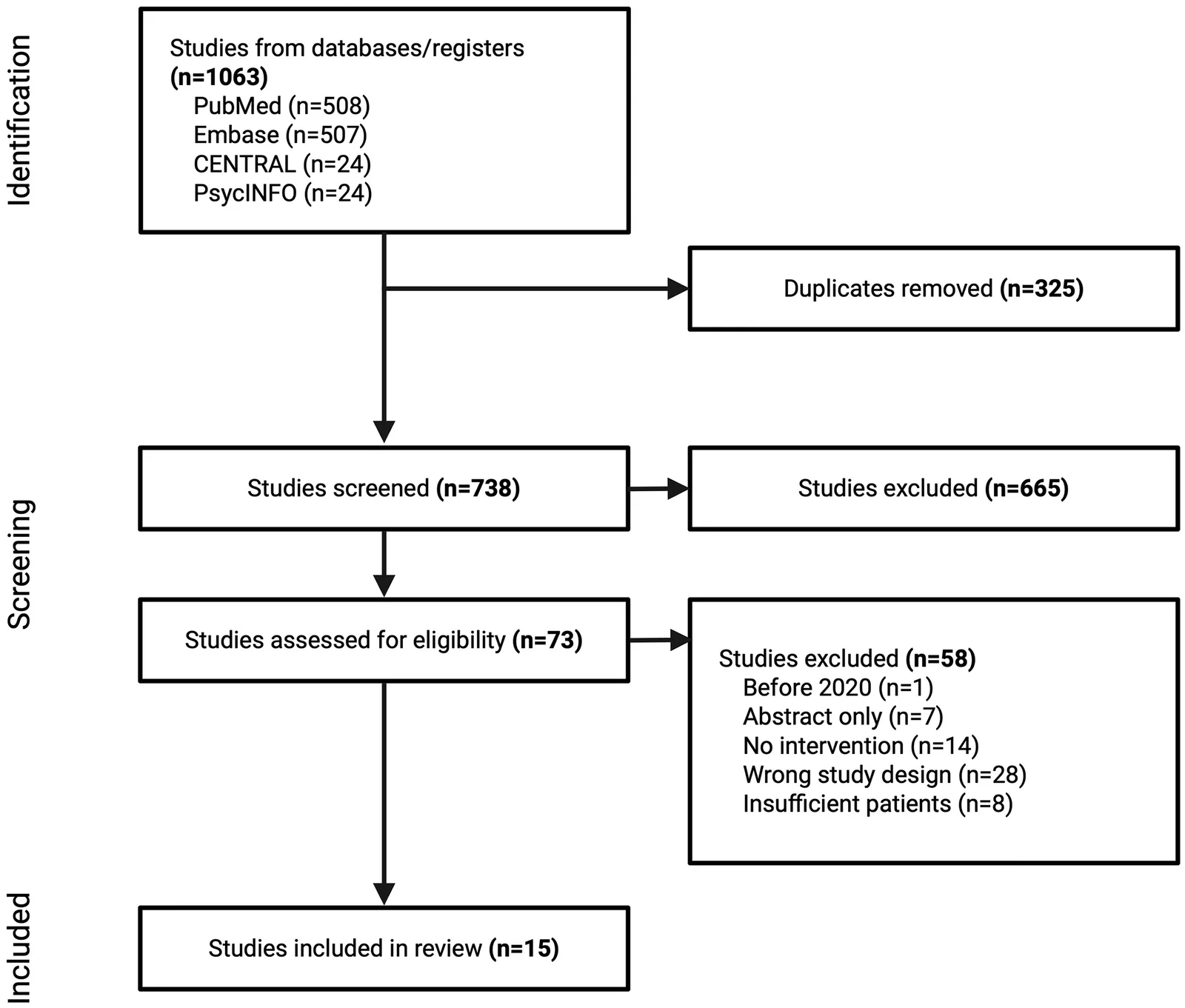
PRISMA flow diagram of systematic review.

Duplicates and analogous terms were merged to form 68 unique outcomes. The most commonly reported outcomes were the improvement in hydration and volume status, the improvement in nutritional status and stoma prolapse as a complication of treatment.

Eleven further unique outcomes were generated through four 90-min online patient focus groups (led by RB). The study management group added a further nine. The resultant 88 unique outcomes made up the “long list” of outcomes to enter round 1 of the online Delphi process. A summary of these outcomes, along with their lay definitions, organized by theme, is presented in Supplementary Table 1.

### 5.3. Outcome prioritization

#### 5.3.1. Round 1

The 88 outcomes were reviewed by participants in round 1. In total, 47 outcomes were excluded according to the preset criteria. A further six *de novo* outcomes were added to the list of outcomes following feedback from the participants at the end of round 1. The study management group then considered the list of outcomes, along with the feedback from the participants, and consolidated the outcomes to a list of 38 outcomes to be included in round 2.

#### 5.3.2. Round 2

Of the 38 outcomes that entered round 2, 37 were deemed to be “critically important” by the participants. Feedback was also provided and a further 12 outcomes that were excluded in round 1 were suggested by the participants to be included in the next round. Following consideration of this feedback, a total of 37 outcomes were carried forward to the consensus meeting (see Supplementary Table 2).

### 5.4. Consensus meeting

Following scoring at the consensus meeting in 2024, six outcomes were retained in the final COS. This constituted two patient-reported and four clinician-reported outcomes (Table 2).

**Table 2.** Final core outcome set.

Theme	Core outcome
**Impact on volume status & hydration**	• Maintenance of hydration and volume status
**Impact on nutritional status**	• Maintenance of nutritional status
**IBD-specific outcomes**	• Clinical activity: based on symptoms • Disease activity: based on objective markers
**Impact on quality of life**	• IBD-related quality of life • Stoma-specific quality of life *QoL outcomes should incorporate all the following domains and be kept separate:* *Restriction in ability to go to work or school (including work at home)* *Restriction in social activities* *Restriction in diet* *Restriction with clothing* *Improvement in fatigue* *Improvement in sexual function or intimacy* *Impact on reproduction*
**Adverse effects from treatment**	• Not included in core outcomes (no consensus achieved) • Complications were considered important to capture (where applicable)—eg, surgical intervention

The outcome “adverse effects from treatment” was felt to be important by the participants at the consensus meeting but did not meet the criteria for retention in the COS after the consensus discussion and voting. Participants’ main reason for excluding this outcome was that adverse outcomes from treatment were heterogeneous, might only be applicable to a specific surgical intervention, and difficult to measure in a core outcome set. The attendees agreed that adverse effects of treatment should be reflected and described in studies where it was relevant and feasible to do so.

At the consensus meeting, it was felt that impact on quality of life was reflected by “IBD-related quality of Life” and “Stoma-specific quality of Life” separately with 83% consensus. Individual outcomes in the quality-of-life theme did not meet universal consensus. However, members of the consensus meeting felt that their inclusion was important but were unable to differentiate the importance between them. After a unanimous decision, it was decided that the individual domains (see COS, Table 2) should be separately incorporated in the quality-of-life outcomes. The progression from long-list development through Delphi rounds to final consensus is summarized in Figure 3.

**Figure 3.**
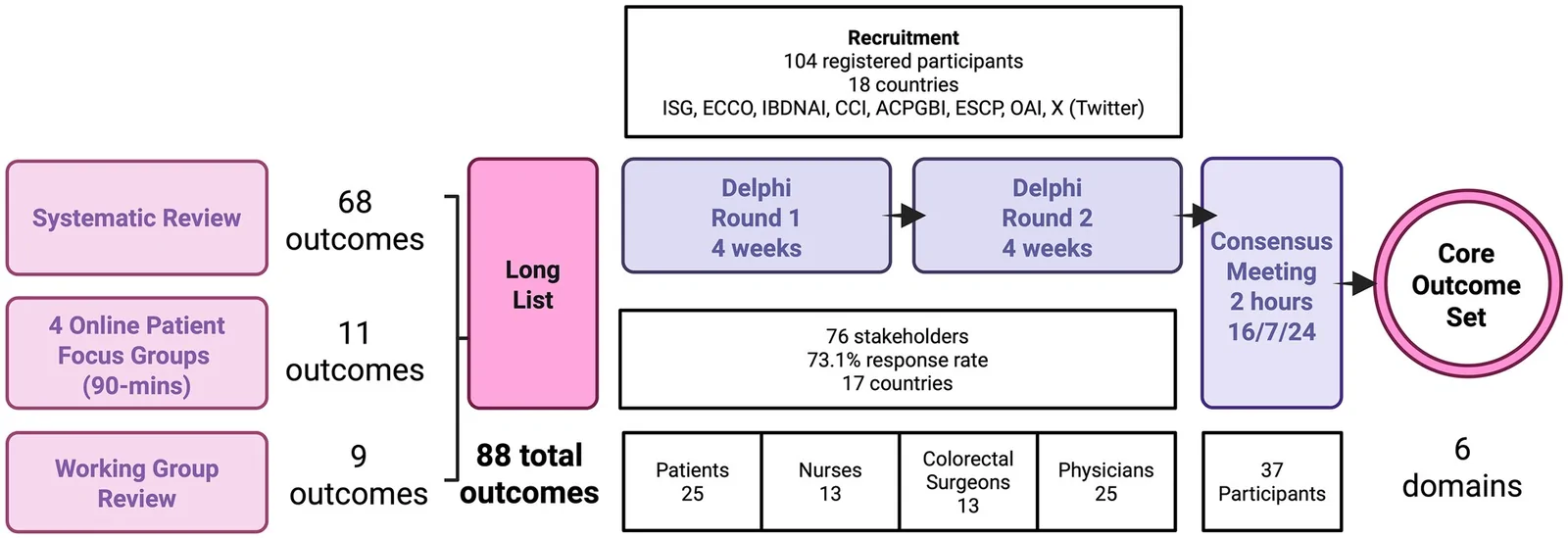
Project flow chart (results).

## 5. Discussion

This is the first study to standardize outcome reporting in IBD patients with stomas and follows the recommendations of the COMET Initiative for COS development. An exhaustive list of candidate outcomes was generated through a systematic review of contemporary outcomes, along with patients’ input from structured focus groups. By using an online tool, a large number of stakeholders from various countries were able to participate in a pragmatic Delphi consensus process that emphasized clinical relevance as well as patient perspective. Through consensus voting and discussion, a COS which is applicable to all study designs was generated with stakeholder support.

As a result, it has been possible to establish a six-domain COS that can be used for clinical effectiveness research evaluating interventions in IBD patients with stomas. Furthermore, the COS outlines important outcome domains to be integrated into the quality-of-life outcomes in this patient population.

Having an ostomy in the setting of IBD denotes a severe disease course, refractory to medication or a consequence of disease-related complications.^34^ Furthermore, a significant proportion of patients with IBD, especially those with CD, and an ostomy are at subsequent high risk of recurrence (typically in the bowel proximal to their stoma).^35^ This cohort of patients represents a unique subgroup of patients who experience not only stoma-related complications but also disease-related complications.^36^ Building a COS begins the process of addressing this unmet need in IBD research.^37^^,^^38^

Following development of this COS, measurement instruments for each of these outcomes will need to be created or modified if not readily applicable to this specific cohort and will likely involve several participants from our study to assist with this. These outcome measurement instruments (OMIs) along with our COS will then be prospectively validated on IBD patients with stomas.

In our systematic review, 53 specific measures were identified, though not all these measures fell under the six domains of the COS. Hydration and volume status appeared in four studies and was assessed using measures including 24-h stoma output (recorded by volume or weight), percentage reduction in stoma output, urine output, urinary sodium, plasma renin and aldosterone, requirement for oral or intravenous fluids, dehydration-related readmission, acute kidney injury, and parenteral-support volume. Nutritional status appeared in three studies but was represented by 24 measures including body weight, body mass index, laboratory tests, and subjective clinician-rated nutritional improvement. Similar heterogeneity in outcome measures was seen in the remaining domains of the COS. Even within a domain, differences in parameters such as collection method, metric, and assessment time prevented direct comparison. Contemporary objective measures such as standardized endoscopic indices or radiological markers were largely absent. The extracted measures, therefore, provide a candidate pool for development of a core measurement set but their frequency of use would not be sufficient evidence of validity or suitability in adults with IBD and a stoma.

By identifying outcomes that are important to both patients and healthcare providers, resources can be appropriately allocated to define the best outcome measures or to build the necessary OMIs for these outcomes. The COS is not aimed to be exhaustive but reflects the minimum outcomes that need to be included and measured in future intervention trials. It does not limit additional outcomes being assessed in clinical studies and we acknowledge that future iterations of this COS could incorporate additional outcomes and outcome measures if deemed to be relevant to patients and stakeholders. The development of a core measurement set to complement this COS is also now underway.

A strength of this study is that every stage of this project, including the addition and exclusion of outcomes as well as the rationalizing of the outcome list, was performed by consensus and always included patient representation. Our study also places an emphasis on using as many online tools as possible (eg, online patient focus groups, online Delphi consensus, online final consensus meeting), maximizing our international reach for this project, reducing the environmental impact of the study (by avoiding the need for travel), as well as making the process convenient for our participants, thereby minimizing attrition where possible. Another strength lies in the inclusion of a diverse study management group and the inclusion of relevant stakeholders from different geographic locations. This meant that the outcomes included in the COS not only considered viewpoints from the different parties involved in the care of IBD and ostomies but also accounted for the possible cultural differences and approaches from different healthcare systems. This is especially relevant to the quality-of-life outcomes and the included domains in this COS.

By using an online-only approach, we do acknowledge the limitation that a small number of participants who were unfamiliar with these IT tools may not have been represented in the project. Additionally, a stakeholder not included in this process was industry; this was excluded due to the potential of bias but we felt would have been important to involve in the adoption and promotion of this COS.

## 6. Conclusion

Through a thorough and systematic approach, with representative stakeholders involved, we have developed a COS for use in studies involving IBD patients with stoma. Groups assessing treatment in this cohort of patients are strongly encouraged to adopt and use this COS to reduce heterogeneity of outcome reporting and improve the quality and comparability of studies in the future.

## Supplementary Material

jjag157_Supplementary_Data

## Data Availability

Data obtained from the Systematic Review, Patient Focus Groups, and Delphi Process were accessible for analysis by the Project Steering Group. Summary data (eg, question responses, eliminated and included outcomes from each round) were made available to the entire working group and are also be part of the supplementary data.
