# Supplementary material for: Development of a core outcome set for IBD patients with stomas
Source: J Crohns Colitis. 2026 Oct 28;20(10):jjag157. doi: 10.1093/ecco-jcc/jjag157 (PMC13716488; doi:10.1093/ecco-jcc/jjag157)
Supplement: Supplementary file 1 [file jjag157_supplementary_data.zip › Supplementary Table 2.pdf]

Consensus Meeting Outcome List

No.	Theme	Outcome
1	**Impact on Volume Status & Stoma Activity**	Maintenance of hydration and volume status
2		Improvement in stoma frequency
3		Reduction in stoma leaking or breaks in bag due to overactivity
4	**Impact on Nutritional Status**	Maintenance of nutritional status
5		Bone health
6	**IBD Specific Outcomes**	Improvement in blood-based inflammatory markers (e.g. CRP)
7		Improvement in stool-based inflammatory markers (e.g. FCP)
8		Improvement in endoscopic findings
9		Improvement in histological inflammation
10		Improvement in radiological findings (e.g. MRI/Intestinal Ultrasound)
11		Improvement in extra-intestinal manifestations of IBD
12		Prevention of clinical recurrence of IBD
13		Reduced recurrence of IBD-related stomal obstruction
14	**Impact on Quality of Life**	Global quality of life
15		IBD Quality of Life Status
16		Stoma Quality of Life status
17		Faecal Incontinence Quality of Life status
18		Restriction in ability to go to work or school
19		Restriction in social activities
20		Restriction in family relationships
21		Restriction in diet
22		Improvement in sexual function or intimacy
23		Improvement in fatigue
24		Restriction in clothing
25		Confidence with stoma function
26		Impact on reproduction
27	**Adverse Effects from Treatment**	Stoma Complication: Stoma leak
28		Stoma Complication: Parastomal hernia
29		Stoma Complication: Stoma obstruction
30		Surgical complication: 30-day mortality
31		Surgical complication: 30-day reoperative rate
32		Surgical complication: 30-day readmission
33		Surgical Complication: Major complications (Clavien-Dindo>3)
34		Surgical Complication: Haemorrhage
35		Surgical Complication: Wound Infection
36		Surgical Complication: Port-site incisional hernia
37		Infection
