# Supplementary material for: Development of a core outcome set for IBD patients with stomas
Source: J Crohns Colitis. 2026 Oct 28;20(10):jjag157. doi: 10.1093/ecco-jcc/jjag157 (PMC13716488; doi:10.1093/ecco-jcc/jjag157)
Supplement: Supplementary file 1 [file jjag157_supplementary_data.zip › Supplementary File 1.docx]

**Summary of Results from Round 1**

Number of Participants: 104

Countries: 18

Panel 1 (Patients): 35

Panel 2 (Gastroenterologists): 31

Panel 3 (Colorectal Surgeons): 20

Panel 4 (Nurses): 18

**Patient Demographics:**

Age (Range): 25-72 years

Gender: 24 (F), 21 (M)

Disease: 22 Crohn’s, 13 UC

Type of Stomas:

• 33 x Ileostomy, 2 x Colostomy

• 31 x Permanent, 4 x Temporary

Number of IBD-related surgeries (Range): 1-10

Duration of IBD (years, range): 1-40 years

12/35 (34.2%) have undergone revision surgery for their stoma

8/35 (22.9%) have undergone previous pouch forming surgery

9/35 (25.7%) still have a rectum in-situ

24/35 (68.6%) have experienced a flare of IBD since stoma formation

16/35 (45.7%) are currently on medication for IBD

**Outcomes (Start of Round 1):**

Total: 88

Impact on Volume Status & Hydration: 9

Impact on Nutritional Status: 4

IBD Specific Outcomes: 11

Impact on Quality of Life: 20

Adverse Effects from Treatment: 44

**Summary of Results from Round 2**

Number of Participants: 76

Countries: 17

Panel 1 (Patients): 25

Panel 2 (Gastroenterologists): 25

Panel 3 (Colorectal Surgeons): 13

Panel 4 (Nurses): 13

**Outcomes (Start of Round 2):**

Total: 38

Impact on Volume Status & Hydration: 3

Impact on Nutritional Status: 2

IBD Specific Outcomes: 9

Impact on Quality of Life: 13

Adverse Effects from Treatment: 11
