# Supplementary material for: Development of a core outcome set for IBD patients with stomas
Source: J Crohns Colitis. 2026 Oct 28;20(10):jjag157. doi: 10.1093/ecco-jcc/jjag157 (PMC13716488; doi:10.1093/ecco-jcc/jjag157)
Supplement: Supplementary file 1 [file jjag157_supplementary_data.zip › Supplementary Table 1.pdf]

Theme	Outcomes	Lay Definitions (Outcomes)	Studies	No. of Studies
**Impact on Volume Status and Stoma Activity**	Improvement in hydration	An improvement in how much fluid there is in the bloodstream to support normal bodily functions	1,4,5,8	4
	Resolution of volume depletion	Resolving an excess loss of fluid from the body	1,4,5,8	4
	Improvement in stoma frequency	An improvement in how much stool comes through the stoma	FG	
	Reduction in stoma flatus	Reduction of how much gas comes through the stoma	FG	
	Improvement in stool consistency from stoma	More solid stool seen in stoma	FG	
	Reduction in stoma leaking or breaks in bag due to overactivity	Reduction in leaks or breaks in the bag due to the bag overfilling	FG	
	Improvement in symptoms related to dehydration e.g. dizziness, headaches, thirst	Improvement in symptoms associated with being dehydrated	WG	
	Resoluton of Constipation	No difficulty in passing stool with regular soft bowel motions via stoma	10	1
	Independence with stoma (volume and hydration)	Being able to maintain a good level of hydration without needing external support (e.g. hospitalisation, home care)	WG	
**Impact on Nutritional Status**	Improvement in nutritional status	An improvement in nutritional status	1,5,6	3
	Anorexia	Not consuming food due to a lack of desire to eat	FG	
	Improvement in appetite	An increased desire to eat	FG	
	Independence with stoma (nutrition)	Being able to meet nutritional needs without external support (e.g. hospitalisation, home care)	WG	
**IBD Specific Outcomes**	Improvement in serological inflammatory markers	An improvement in specific blood tests that assess for inflammation	5	1
	Improvement in histological inflammation	An decrease in inflammation seen in biopsy samples when assessed under a microscope	5,9	2
	Improvement in stool-based inflammatory markers	An improvement in specific stool tests that assess for inflammation	7	1
	Improvement in radiological findings	Reduction in severity of inflammation seen on imaging - e.g. MRI scan of the bowel or Ultrasound scan of the bowel	WG	
	Improvement in microbiome analysis	An improvement in the bacterial composition of the gut (fewer bacteria associated with inflammation)	WG	
	Improvement in endoscopic findings	Reduction in severity of inflammation seen during endoscopy	WG	
	Improvement in extra-intestinal manifestations of IBD (e.g. arthralgia, cutaneous manifesations)	An improvement in non-bowel related symptoms such as joint stiffness and pain or skin rashes	FG	
	Prevention of clinical recurrence of IBD	Prevention of a recurrence of IBD	9	1
	Reduced recurrence of IBD-related stomal obstruction	Reduced number of times that stoma gets blocked due to inflammation	FG	
	Improvement in IBD-related abdominal pain	Reduced pain and cramps in the tummy caused by active inflammatory bowel disease	FG	
	Resolution of pyoderma gangrenosum	Resolution of a painful, inflammatory skin condition around a stoma	12	1
**Impact on Patient Quality of Life**	Global QOL status	A generalised reflection of one's quality of life	2,3	2
	IBD Quality of Life Status	A scoring system to assess aspects of a person's life specific to Inflammatory Bowel Disease	2,3	2
	Stoma Quality Of Life status	A scoring system to assess aspects of a person's life with respect to their stoma	2,8	2
	Fecal incontinence Quality Of Life status	A scoring system to assess aspects of a person's life with respect to the ability to control bowel movements	2	1
	Performance Status (ability to return to work/school)	Ability to go to work or school	2	1
	Restriction in sports	Unable to partcipate sports activities	2	1
	Restriction in social activities	Unable to leave the house to meet friends, go to parties etc	2,3	2
	Restriction in recreation	Unable to participate in leisurely activties e.g. going for a walk	2	1
	Restriction in work around the home	Unable to perform chores in the house	2	1
	Restriction in family relationships	Unable to stay connected with family members	2	1
	Restriction in travel	Unable to use certain modes of transport e.g. public transport	2	1
	Restriction in diet	Restriction in the amount of food consumed	2	1
	Improvement in sexual function/Reduced restriction in sexual function	A scoring system to assess aspects of sexual function	2,3	2
	Improvement in bowel habit	A scoring system (based on a scale) to look at change in bowel movements	2	1
	Improvement in body image	Feeling better about one's mental image of their own body	2	1

	Improvement in Sleep	Improvement in the amount of sleep at night or fewer disruptions in sleep	FG	
	Improvement in Fatigue	Feeling less tired or feeling more energetic	FG	
	Impact on Financial Burden	Difficulty in managing finances due to time away from work due to illness	WG	
	Impact on Education	Difficulty in progression of education due to time away from school due to illness	WG	
	Changes in patient-reported outcomes	Change in the experiences, symtoms and quality of life described by the patients themselves	5	1
**Adverse Effects of Treatment (Outcomes as a direct result from treatment)**	Stoma complication: Stoma swelling	Swelling of the stoma	1	1
	Stoma complication: Stoma prolapse	The bowel protrudes out too far from the skin	1,5,8,9,13,14	6
	Stoma complication: Stoma retraction	The bowel goes inward or shrinks back towards the body	8,9,14,15	4
	Stoma complication: Fistula	Abnormal connection from the bowel to the skin	8,11,14	3
	Stoma complication: Granuloma	Benign tissue growth around the stoma	8	1
	Stoma complication: Stoma necrosis	Lack of blood supply of the stoma leading to compromise of the health of the bowel tissue	5,8,14,15	4
	Stoma complication: Stoma leak	A breach in the seal or fit of the stoma bag resulting in a leak	3,8,15	3
	Stoma complication: Peristomal skin irritation/ulceration	Skin problems around the stoma	3,8,9,15	4
	Stoma complication: Parastomal hernia	A hernia (a visible bulge or lump) around the stoma	3,8,9,11,14	5
	Stoma complication: Stoma stenosis	Narrowing of the stoma leading to reduced output	8,9,14	3
	Stoma complication: Stoma obstruction	Complete blockage of the stoma	5,10	2
	Surgical complication: 30-day mortality	Death within 30 days of an operation	3,13,15	3
	Surgical complication: 30-day reoperative rate	Requiring a repeat operation within 30 days	3,15	2
	Surgical complication: 30-day readmission	Being admitted to the hospital again within 30 days	3,13	2
	Surgical complication: Prolonged hospital length of stay	Requiring to stay in hospital for longer than expected	3,13	2
	Surgical Complication: Major complications (Clavien-Dindo>3)	Major complications from surgery requiring medication, blood transfusions etc to resolve the issue	3,11	2
	Surgical Complication: Failed ileoanal anastomosis creation	Failure to connect the small bowel to the anus, leaving the patient with a permanent stoma	WG	
	Surgical complication: Failed valve construction	Failure to create a valve from the small bowel for a specific type of stoma	14	1
	Surgical Complication: Ileus	Temporary interruption or paralysis of the movement of the bowel - presenting like a blockage	3,13	2
	Surgical Complication: Haemorrhage	Significant bleeding	8,13,14	2
	Surgical Complication: Abscess	An infected collection of pus	3,13,14,15	4
	Surgical complication: Seroma	Development of a pocket of clear fluid around the surgical site	11	1
	Surgical complication: Wound dehiscence	Separation or opening of the edges of a surgical incision or wound, typically before it has fully healed	3,8,11,13,15	5
	Surgical Complication: Wound Infection	An infection of the surgical wound site	3,11,13,14	4
	Infection	Developing an infection (non-specific) after surgery	8,13	2
	Sepsis	Life-threatening Infection	3,5	2
	Venous Thromboembolism (VTE) - DVT/PE	Blood clot (e.g. in the legs or in the lungs)	3	1
	Acute renal failure	Sudden and rapid loss of kidney function	3,13	2
	Cardiac complications e.g. cardiac arrest, tachycardia	Complications of the heart including stopping of the heart or abnormal heart rhythm	3,5	2
	Respiratory Complications e.g. infection, cough, shortness of breath	Breathing complications including an chest infection, feeling short of breat or a cough	1,5,13	3
	Urinary Complications e.g. infection, retention, polyuria	Complications of the waterworks including a urinary tract infection, inability to pass urine or passing urine too frequently	1,3,5,14	4
	Nausea or Vomiting	Nausea or vomiting	1,5	2
	Heartburn	Acid indigestion or reflux	4	1
	Oedema	Swelling of the ankles	1,5	2
	Skin discolouring	Changes to the colour of the skin	4	1

	Stiffness of interphalangeal joints	Reduced flexibility or reduced range of motion in fingers or toes	4	1
	Hot flushes	Sensation of heat or warmth spreading over the body, associated with redness and sweating	5	1
	Dizziness	Feeling dizzy or light-headed	5	1
	Transient injection site reactions	Short-lived reaction (pain/swelling/redness/itch etc) of the site where the injection was given	5	1
	Fatigue	Feeling extremely tired	5	1
	Decreased appetite	Reduced hunger	5	1
	Diarrhoea	Increased output from stoma	10,13	2
	Unpleasant odor of the stool	Unpleasant smell of stool from stoma	10	1
	Flatulence	Increased wind/gas from stoma	10	1
**Excluded by the WG**	Changes in pharmacokinetic profiles		5	1
	Incidence of anti-glepaglutide antibodies		5	1
	Changes in physical examination evaluations		5	1
	Collapse during ileoscopy		4	1
	Complication: intestinal lesions		14	1
